# Targeting of Rac GTPases blocks the spread of intact human breast cancer

**DOI:** 10.18632/oncotarget.520

**Published:** 2012-06-09

**Authors:** Elad Katz, Andrew H. Sims, Duncan Sproul, Helen Caldwell, J. Michael Dixon, Richard R. Meehan, David J. Harrison

**Affiliations:** ^1^ Breakthrough Breast Cancer Research Unit, University of Edinburgh, Western General Hospital, Edinburgh, United Kingdom.; ^2^ MRC Human Genetics Unit, Institute of Genetics and Molecular Medicine, University of Edinburgh, Western General Hospital, Edinburgh, United Kingdom; ^3^ School of Medicine, University of St Andrews, St Andrews

**Keywords:** Breast cancer, GTPase, Rac1, STAT3, Invasion

## Abstract

High expression of Rac small GTPases in invasive breast ductal carcinoma is associated with poor prognosis, but its therapeutic value in human cancers is not clear. The aim of the current study was to determine the response of human primary breast cancers to Rac-based drug treatments ex vivo.

Three-dimensional organotypic cultures were used to assess candidate therapeutic avenues in invasive breast cancers. Uniquely, in these primary cultures, the tumour is not disaggregated, with both epithelial and mesenchymal components maintained within a three-dimensional matrix of type I collagen. EHT 1864, a small molecule inhibitor of Rac GTPases, prevents spread of breast cancers in this setting, and also reduces proliferation at the invading edge. Rac1+ epithelial cells in breast tumours also contain high levels of the phosphorylated form of the transcription factor STAT3. The small molecule Stattic inhibits activation of STAT3 and induces effects similar to those seen with EHT 1864. Pan-Rac inhibition of proliferation precedes down-regulation of STAT3 activity, defining it as the last step in Rac activation during human breast cancer invasion.

Our data highlights the potential use of Rac and STAT3 inhibition in treatment of invasive human breast cancer and the benefit of studying novel cancer treatments using three-dimensional primary tumour tissue explant cultures.

## INTRODUCTION

In recent years there has been a growing appreciation that developing new effective treatments may be facilitated by experimental systems that replicate the multi-component tumour environment [1, 2]. Furthermore, there are serious doubts regarding the relevance of drug testing in established cancer cell lines and transfer of results to the clinic [3]. We have developed a three-dimensional culture system that allows maintenance of complex breast cancer specimens explanted *ex vivo* for up to four weeks after surgical resection in a supporting matrix of exogenous stromal type I collagen [4, 5]. These *ex vivo* cultures offer a relatively rapid and quantitative avenue to explore mechanisms of tumour spread and evaluate new treatments.

The metastatic potential of cancer cells is orchestrated by cell-cell adhesion, cell-matrix adhesion, protrusion and contractility, all of which require appropriate regulation and dynamics of the cytoskeleton [6]. The Ras-like Rho family GTPases are key regulators of all of these cellular processes due to their interaction with multiple downstream targets. The ability of Rac1 to induce metastasis has been closely linked to mesenchymal type motility in cancer cell lines [7-9]. This is likely to be due to Rac activity near the cell leading edge and subsequent disruption of cellular organization [10, 11]. Nonetheless, Rac activity must be tightly regulated in order to maintain normal epithelial cell junction formation, polarization and proliferation [12-14].

Rac activation can induce invasion and metastasis of breast cancer cell line models *in vitro* and *in vivo* [15]. Over-expression of Rac1 and Rac3 GTPases has been noted in several small cohorts of breast cancers [16-18] and it has been suggested that they may have a role in resistance to endocrine treatment [19, 20]. In breast cancer, this mechanism is best described in the context of HER2 over-expression. As a result of TGFβ stimulation, a complex comprising HER2, Vav2, Rac1, Pak1, actin and actinin is formed at cell protrusions [21]. The induction of Rac1 activity by the guanine nucleotide exchange factor (GEF) Vav2 causes invasion and contributes to cancer cell survival [20] and disruption of correct polarisation of the breast epithelium [22]. Other GEFs may also stimulate Rac1 downstream of HER2, including P-REX1 [22]. Up-regulation of Rac1 activity by P-REX1 and Tiam1 GEFs has been described independently of tumour HER2 status [21], although the latter finding is disputed [23].

Across a panel of 51 established breast cell lines [24], both normal and malignant, similar, detectable levels of Rac1 mRNA were observed. The levels of Rac2 and Rac3 transcripts were more variable, being reliably detected in less than half of the cell lines (our unpublished observations). Studies of Rac function using cell lines already have contributed to understanding of initiation of malignant transformation, but do not inform how intact human tumours may respond to therapeutic intervention [25]. We previously reported that the majority of invasive human breast cancers continue to express E-cadherin and β-catenin correctly at the cell membrane junctions [26]. Therefore, pan-Rac inhibition of the whole, intact breast tumour explant could potentially bring about apparently conflicting effects on epithelial versus mesenchymal cells. It may lead to destruction of epithelial junctions enabling invasiveness, while blocking mesenchymal motility.

In this study we examine the response of authentic primary breast cancers to Rac-based drug treatments. We show that invasive breast cancers are responsive to exposure to EHT1864, a water soluble pan-Rac inhibitor. Consequently, Rac inhibition blocks a key down-stream effector, the Signal Transducer and Activator of Transcription-3 (STAT3) transcription factor. We show that Rac and STAT3 inhibition regimens are effective at blocking tumour invasion *ex vivo*, independently of known clinical biomarkers such as histological grade and ER status.

## RESULTS

### Rac1 is over-expressed in invasive human breast cancer

**Figure 1:**
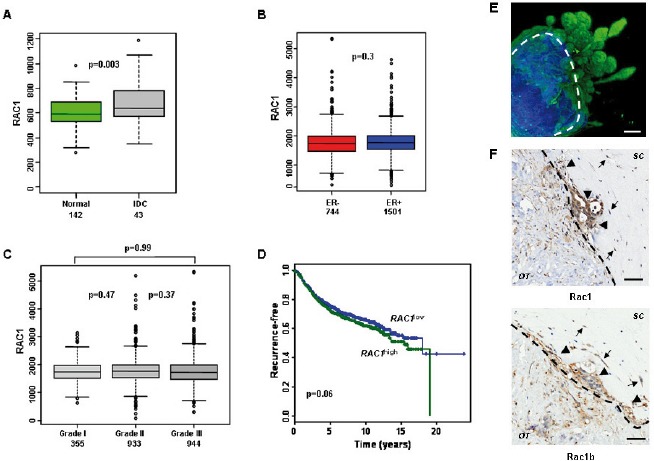
RAC1 is more highly expressed in breast cancer and is associated with poor prognosis (A) Box plots demonstrating that invasive ductal carcinomas (IDC) have higher *RAC1* expression than normal breast ducts, but range is similar. (B-C) *RAC1* is not associated with estrogen receptor alpha (ER) status or histological grade of primary breast tumours. (D) Kaplan-Meier survival curves for all breast cancer patients with available data in the meta-dataset show that high levels of *RAC1* expression are associated with poorer prognosis (n=1654). *RAC1* expression levels were divided into low and high groups at the median. (E) An example of invasion *ex vivo*: 3D reconstruction of optical projection tomography of a representative ER+ HER2- tumour. The original tumour material is in blue (autofluorescence) and invading epithelial tumour cells are in green (cytokeratin labelled). (F) Immunohistochemistry showing that cells invading *ex vivo* express Rac1 protein (*upper panel*) and its short isoform Rac1b (*lower panel*). Rac1 and Rac1b are expressed in both tumour epithelial (*arrowheads*) and mesenchymal (*arrows*) invading cells. Images are from a representative ER- HER2- tumours. Lower magnification images are in Figure S2. Bars, 50 μm. The dotted lines show border between original tumour explant (OT) and surrounding collagen (SC). Brown staining indicates cells positive for Rac1 and Rac1b staining.

In order to determine whether Rac1 protein might be a viable therapeutic target in breast cancer, we analysed its mRNA expression levels in published gene expression datasets. *RAC1* was found to be significantly higher expressed in breast tumours than in normal breast tissue (Figure 1A). We then used a meta dataset of 2999 primary breast tumours (see Methods) to explore whether *RAC1* expression associated with particular subtypes of breast tumours. There was no significant variation in *RAC1* levels with grade or ER status (Figure 1B-C). *RAC1* was significantly higher and associated with poor prognosis in HER2 (*ERBB2*) over-expressing tumours, although the range of *RAC1* expression was similar between HER2^+^ and HER2^−^ tumours (Figure S1). Importantly, high levels of *RAC1* were associated with late recurrence (Figure 1D). Overall, these data suggest that *RAC1* may represent a therapeutic target with broad potential in breast cancer, because it does not show a strong association with particular patient groups.

We have recently succeeded in establishing a robust system for long-term culture of human breast cancer explants, successfully growing >90% tumours of all major sub-types [4, 5]. In these cultures, primary breast cancer biopsies from invasive breast cancers are explanted into the centre of a type I collagen matrix. From each tumour cultured *ex vivo*, 80%-100% of explants are viable and display variable degrees of cellular spread into the surrounding collagen [4]. Growth in mammary epithelial media occurs in all three dimensions, as demonstrated optical projection tomography (Figure 1E; cytokeratin labels epithelial structures (green) invading from the original biopsy material into the surrounding collagen). We found that in many breast cancers grown *ex vivo*, both Rac1 and its constitutively active isoform Rac1b, are preferentially expressed in invading cells (Figures 1F and S2; *n*=6) when assessed by immnunohistochemistry. Importantly, both isoforms are expressed in tumour epithelial and mesenchymal cells, indicating wide spread Rac activity.

### Rac inhibition blocks the spread of human breast cancer

**Figure 2:**
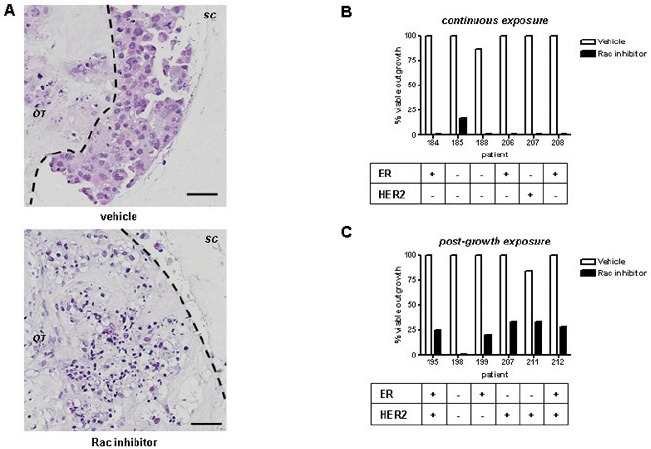
Rac inhibition ex vivo blocks tumour invasion (A) Tumour invasion into surrounding collagen is readily detectable using H&E staining (*upper panel*). Continuous Rac inhibition with EHT1864 for 14d (*bottom panel*) results in blockage of invasion beyond the original tumour material (black outline) and extensive cell death: Bars, 50 μm. Outgrowth of tumour cultures were categorised following treatment with EHT 1864 or vehicle control, as follows: (B) continuous exposure from start of culture, or (C) exposure of existing outgrowths, 10d after start of culture. All inhibitor treatments lasted 14d. Results were obtained from light microscopy examination and subsequently confirmed by H&E staining. Tumour biomarkers shown were determined by a pathologist as part of clinical practice.

We used EHT 1864, a water soluble pan-Rac inhibitor [27], to determine the value of Rac inhibition in the explant model. EHT1864 exhibits high affinity binding to Rac1, as well as the related Rac1b, Rac2, and Rac3 isoforms, inhibiting their activity via a mechanism that involves guanine nucleotide displacement [27]. EHT 1864 has been used previously in cell line models in 2D, especially in the context of Rac1 activity [19, 28]. Continuous treatment of breast cancer explant cultures with EHT 1864 for 14 days resulted in complete block of invasion into the surrounding collagen, and wide spread cell death (Figure 2A). We characterised the effects of Rac inhibition in two regimens: in the first regimen, explants from the same cancer were divided randomly to equally sized treatments groups and treated continuously for 14 days (Figure 2B). In the second regimen, explant cultures were left to grow out for 10 days, and then divided randomly to treatment groups for a further 14 days (Figure 2C). Both regimens resulted in almost complete elimination of tumour outgrowth into the surrounding collagen. Using logistic regression, Rac inhibition with EHT 1864 exhibited an odds ratio of 0.01 of outgrowth compared with vehicle (p < 0.0001, 95% CI 0.003 to 0.04).

### Rac inhibition blocks proliferation and induces apoptosis in human breast cancers

**Figure 3:**
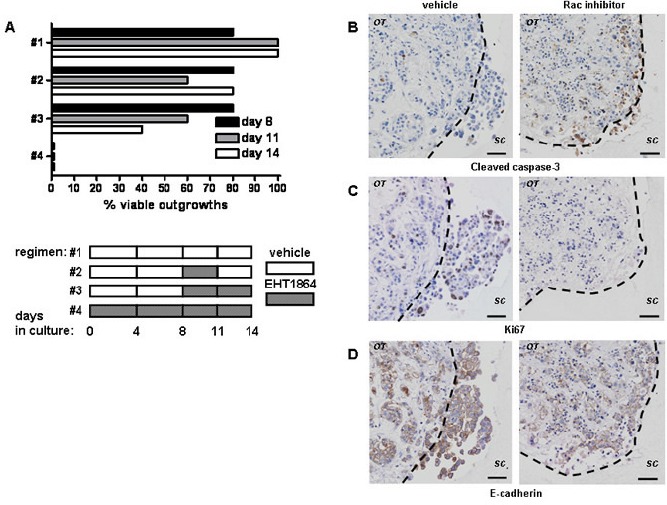
Rac inhibition with of tumour outgrowth ex vivo results in block of proliferation and cell death (A) The effect of treatment with EHT 1864 or vehicle control on outgrowth of tumour cultures was obtained from light microscopy examination and subsequently confirmed by H&E staining. Shown are reperentative results from ER- HER2- tumour explant culture lasting 14d (*n*=4). Note that continuous EHT1854 treatment reduces viable outgrowth (regimen #4) in comparison to short-treatment (#2) or vehicle control (#1). Rac inhibition of tumour invasion is accompanied with induction of apoptosis as detected by immunohistochemistry for cleaved caspase-3 (B) and block of proliferation, detected by Ki67 staining (C). (D) E-cadherin levels are unchanged *ex vivo*. Shown here representative images from a single ER- HER2- tumour, (*left panel*) without or (*right panel*) with EHT 1864. The dotted lines show border between original tumour explant (OT) and surrounding collagen (SC). Bars, 50 μm.

Tumour outgrowth resumed if Rac inhibition was removed after 3 days of treatment (Figure 3A; 4 independent experiments), indicating that EHT 1864 treatment is not solely as a result of cytotoxicity. We therefore examined what down-stream effects on apoptosis (assessed by demonstration of cleaved caspase-3) and proliferation (as assessed by Ki67). The number of cells undergoing cell death at the experimental endpoint rose from a basal level of <20% in vehicle-treated tumours [5] to 30%-50% of cells treated with EHT 1864. These cells were found predominantly around the periphery of the original tumour explant (Figure 3B). Vehicle-treated tumours have a wide range of proliferation from 10% to 60% of cells [5], but exposure to EHT 1864 reduced proliferation to less than 5% (Figure 3C). Interestingly, levels of E-cadherin expression were very similar with or without EHT 1864 treatment (Figure 3D).

### STAT3 is a down-stream mediator of Rac activity in breast cancer spread

A cell line 3D model was established to assess the potential contribution of Rac1 down-stream targets in epithelial cell invasion. We modified our collagen-based invasion culture [29] using the HCC1954 cell line, which invades in organised fashion *in vivo* [30]. In 3D culture, HCC1954 cells express proteins such as E-cadherin in a similar manner to this observed in tumour cells cultured *ex vivo* (Figure S3). In the absence of serum, HCC1954 cells invade collectively (Figure S4A). Treatment of the cell line culture with EHT 1864 resulted in rapid (<24 h) inhibition of invasion and appearance of apoptotic cells (Figures S5A). Surviving HCC1954 cells were still positive for Ki67, a marker of proliferation (Figure S5B). The effects of pan-Rac inhibition in the cell line model could not be replicated by inhibitors against many known Rac downstream mediators: JNK (SP600125), PAK (IPA3) or p38α (SB203580) (data not shown).

**Figure 4:**
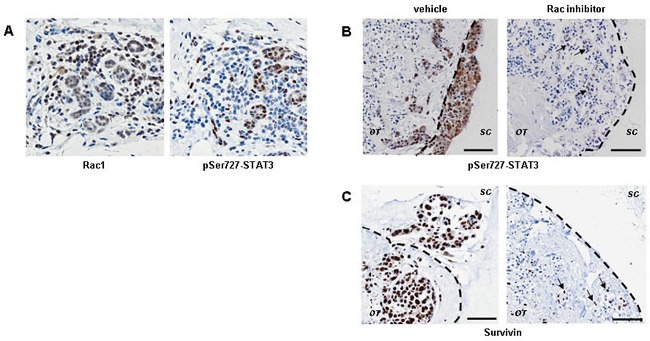
STAT3 signaling is downstream of Rac activity during invasion of human breast cancer (A) Immunohistochemistry shows that Rac1 protein expression (*left panel*) and STAT3 phosphorylation on Ser727 (*right panel*) frequently coincide in human breast cancer. Epithelial cells which are Rac1+ pSTAT3- are also present. (B) STAT3 phosphorylation is associated with invasion *ex vivo* and is down regulated by Rac inhibition (*right panels*; inhibition as in Figure 2). Identical patterns are seen with the STAT3 transcriptional target Survivin, *ex vivo* (C). Even after exposure to EHT 1864, a few cells are still positive for phospho-STAT3 and Survivin (arrows in B and C). Images are shown from a representative ER- HER2- tumour. Lower magnification images are in Figure S6. The dotted lines show border between original tumour explant (OT) and surrounding collagen (SC). Bars, 50 μm.

Another well established Rac1 target is phosphorylation of STAT3 at Ser727 is [31]. We examined Rac1 and phospho-Ser727 STAT3 levels by antibody staining in human breast tumours (*n*=6). Patterns Rac1 and phospho-Ser727 STAT3 staining are similar, especially in epithelial cells (Figure 4A). Phospho-Ser727 STAT3 staining is abundant in the invading cells *ex vivo* (Figure 4B and S6A). In the HCC1954 model, Rac inhibition resulted in reduction in phospho-Ser727 STAT3 (Figure S6A). Survivin, a well established target of STAT3 transcriptional activity [32], is also down-regulated by EHT 1864 treatment of cell line cultures (Figure S7B). Some HCC1954 cells survived Rac inhibition for 24 h, retaining both phospho-Ser727 STAT3 and Survivin (Figure S5, arrows).

### Block of proliferation by Rac inhibition *ex vivo* precedes down-regulation of STAT3 activity

We tested whether long-term Rac inhibition also blocks STAT3 activity in the explant tumour cultures. The intensity of phospho-Ser727 STAT3 staining *ex vivo* was greatly reduced by EHT 1864 (Figure 4B). Consistent with STAT3 activity down-stream of Rac, EHT 1864 treatment resulted in down-regulation of Survivin protein expression (Figures 4C). Interestingly, cells initially surviving Rac inhibition retained both nuclear localization of phospho-Ser727 STAT3 and Survivin *ex vivo* (arrows).

**Figure 5:**
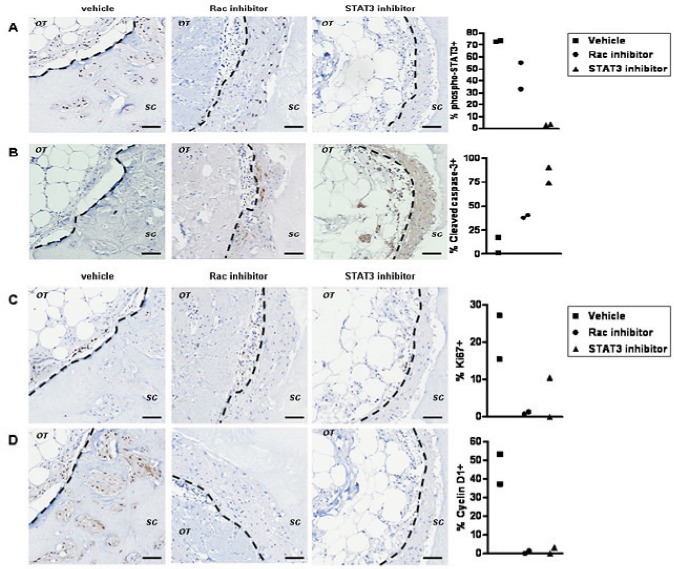
The early cellular response to Rac inhibition ex vivo (A) Tumour explants were grown *ex vivo* for 14 days prior to inhibitor treatment in order to define events induced by a single post-growth treatment. Immunohistochemistry suggest that STAT3 phosphorylation (vehicle, *left panels*) is down regulated by EHT 1864 (*middle panels*) or Stattic (*right panels*) within 72 h of treatment. (B) Wide spread apoptosis is detected in Stattic treated cultures (*right panel*) and to lesser extent in EHT 1864 treated cultures (*middle panel*). Effects on proliferation are shown by staining with the marker Ki67 and (C) the cell cycle protein cyclin D1 (D): reduced in EHT 1864 (*middle panel*) or Stattic (*right panel*) exposed cultures). The dotted lines show border between original tumour explant (OT) and surrounding collagen (SC). Bars, 50 μm. Quantification is shown for percent of positive invading cells from two preparations per treatment, all originating from the same tumour.

STAT3 may be indirectly controlled by Rac via an autocrine or paracrine signal [33, 34]. We examined if there are short-term effects of Rac inhibition preceding changes in STAT3 activity *ex vivo*. The role of STAT3 downstream of Rac activation was confirmed using Stattic, an inhibitor of dimerization and nuclear translocation of STAT3 preventing its constitutive activation [35]. After one round of treatment with EHT 1864, only a partial reduction in phospho-Ser727 STAT3 and Survivin staining can be observed by immunohistochemistry (Figure 5A and not shown). The rapid induction of apoptosis by STAT3 inhibition in comparison to Rac inhibition, supports a more direct effect of STAT3 inhibition (Figure 5B). Importantly, proliferation within the invading cells is greatly reduced by both inhbitors (Figure 5C), suggesting most likely through down-regulation of cyclin D1 (Figure 5D), a known Rac1 and STAT3 target [36-39]. The dramatic induction of apoptosis by Stattic is even more pronounced than this caused by EHT 1864, in line with previous reports in breast cancer cell lines [35, 40-42].

**Figure 6:**
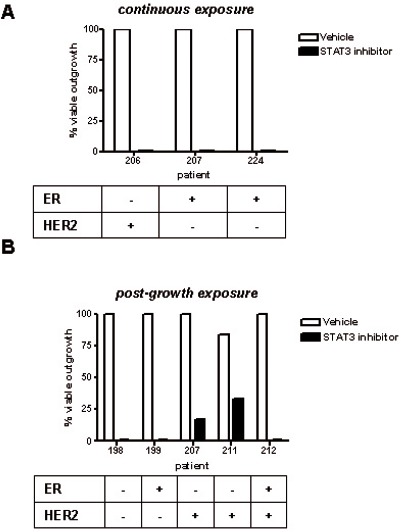
STAT3 inhibition blocks tumour outgrowth ex vivo Outgrowth of tumour cultures were categorised following treatment with Stattic or vehicle control, as follows: (A) continuous exposure from start of *ex vivo* culture, or (B) exposure of existing outgrowths, 10d after start of culture. All inhibitor treatments lasted 14d. Results were obtained from light microscopy examination and subsequently confirmed by H&E staining. Tumour biomarkers shown were determined by a pathologist as part of clinical practice.

Stattic treatment has similar long-term effects to those seen with EHT 1864 on tumour invasion *ex vivo* (Figure 6). Using logistic regression, STAT3 inhibition with Stattic exhibited an odds ratio of 0.003 (p < 0.0001, 95% CI 0.001 to 0.018) compared with vehicle.

## DISCUSSION

Although endocrine (for ER+ tumours), cytotoxic chemotherapy (for ER- tumours) or Herceptin (for HER2^+^ tumours) regimens are successful treatments for some breast cancer patients, mortality due to recurrence of cancers which develop drug resistance and metastases is still common [43]. Therefore, identification of novel treatment for metastatic breast cancer is important to enhance long term patient survival [44]. As direct measurements of enzymatic activity in human tumours are difficult and possibly inaccurate due to clinical constrains in sample collection, other methods are need to evaluate such novel treatments.

The over-expression of Rho GTPases has previously been implicated in the promotion of various cancer types [16, 45]. It is also well established that Rho, Cdc42 and particularly Rac1 have critical roles in experimental tumour metastasis [33, 38]. It is usually thought that Rac activity in cancer is associated with the loss of epithelial cell-cell junctions and gain of mesenchymal phenotype [46]. Many signaling pathways lie downstream of Rac proteins, although their relationship to malignancy is not always clearly understood. These include the activation of JNK and p38 proteins [47], as well as non-canonical (β-catenin-independent) WNT signaling pathways [48]. It has been suggested that the constitutively active isoforms Rac1b and Rac3 show pathway preference, which is distinct from that of the GEF-dependent isoforms Rac1 and Rac2 [8, 16, 49].

Here, we have shown that the spread of breast tumours in an *ex vivo* model is sensitive to Rac inhibition. This 3D culture model recapitulates epithelial invasion of human breast cancer tumours *in vivo*, in particular maintaining E-cadherin membrane expression, unlike invasive breast cancer cell lines [26, 29]. Recent evidence links proliferation and invasion to the spread of cancer, both of which are inhibited in our model system upon EHT 1864 exposure [33, 50]. Moreover, a study in mouse melanoblasts suggested a role for Rac1 in proliferation of motile cells, although the exact mechanism was not identified [51].

Our experiments demonstrate that an approach involving pan-Rac inhibition has the potential to be applied to the clinic. In the current study, inhibition of all Rac isoforms by EHT1864 in explants of intact primary cancers prevented spread into collagen, reduced proliferation and subsequently caused apoptosis. The reduction in cyclin D1 levels by Rac inhibition did not result in greater invasiveness, as reported in MDA-MB-231 cells [52]. EHT 1864 blocks the constitutively active Rac1b and Rac3 isoforms [27], unlike the more widely used Rac GEF inhibitor NSC23766. EHT 1864 is likely to be superior to using a Rac GEF inhibitor, which will not have any effect on the Rac1b or Rac3, long considered to be associated with breast cancer aggressiveness [8, 16]. To date, a range of *in vitro* and *in vivo* models has not reported any significant cytotoxicity associated with EHT 1864. This represents a therapeutic opportunity as breast cancers should have higher over-all Rac activity than this of the surrounding normal breast.

We have demonstrated, at the breast cancer tissue level, a link between STAT3 and Rac proteins. STAT3 is a critical transcription factor regulating cell death during breast involution [53], but it can also function as an oncogene [39]. STAT3 activity (in absence of STAT5 activity) has been shown previously to be present in 40% of human breast cancers and has been linked to higher grade and lymph node metastasis [54]. STAT3 inhibition has a clear anti-tumour effect as demonstrated by a variety of reagents [42, 55]. It is conceivable that disruption of the feedback loop between Rac1 and E-cadherin adherens junctions could lead to aberrant STAT3 activation in breast cancer [56].

The current study suggests that invasion of breast cancer is associated with up-regulation of Rac and STAT3 activity. The challenge now is to understand which breast cancer patients may be most suited for Rac-based treatment, once a drug is available for human use. Our finding that patients with *HER2*^high^ *RAC1*^high^ tumours have the worst prognosis, suggests that Rac targeting in the clinic may particularly benefit patients with HER2^+^ tumours. This is supported by recent experiments in mice suggesting that gp130/STAT3 signalling is critical in HER2-induced tumourigenesis [57]. Our findings that even after exhaustive Rac inhibition in intact breast tumour, STAT3 activity is detected in rare live cells suggest that optimal treatment may require a combination of drugs inhibiting Rac and STAT3 activities.

## MATERIALS AND METHODS

### Gene Expression Analysis

Raw .cel files from seventeen Affymetrix U133A/plus 2 gene expression datasets were downloaded from NCBI GEO (GSE12276, GSE21653, GSE3744, GSE5460, GSE2109, GSE1561, GSE17907, GSE2990, GSE7390, GSE11121, GSE16716, GSE2034, GSE1456, GSE6532, GSE3494 and GSE19615) or the caBIG (geral-00143) repositories, summarised with Ensembl alternative CDF [58] and normalised with RMA [59], before integration using ComBat [60] to remove dataset-specific bias as previously described [61]. The most conservative measure available of recurrence-free, disease-free and distant-metastasis-free survival was used. Cox proportional hazards regression was performed using SPSS version 14.

### Ethical approval

The use of tissue from invasive breast cancer treated at the Edinburgh Breast Unit at the Western General Hospital was approved by the Lothian Research Ethics Committee (06/S1103/65). The clinical parameters of all tumours used in this study are listed in Table S1.

### Collagen-based 3D cultures

The set-up of collagen type I-based cultures, *ex vivo* and cell line, was described in detail previously [4, 29].

For cell line cultures, 75 μL or 150 μL cell-Matrigel plugs were made using 2 × 10^6^ cells/ml and 5mg/ml Matrigel (BD bioscience), in a U-shaped 96-well plate. After overnight incubation, cell plugs were carefully removed from their 96-well plate and embedded in 1 ml of rat collagen I (1 mg/ml) in a 24-well plate or 2 ml of collagen in a 12-well plate. These cultures were incubated for a further 1 h and then carefully freed from the edges of the well (to allow contraction of the collagen) and supplemented with 1 ml or 2 ml RPMI media (without serum). The cells were then left to invade. Media was changed every 3-5 days. Rac inhibitor (EHT 1864, Tocris #3872, 20 μM) or STAT3 inhibitor (Stattic, Tocris #2798, 10 μM) were added at the last 24 h or 96 h.

For *ex vivo* cultures, breast tumour biopsy materials (cut to 1mm pieces) were explated instead of Matrigel plugs in a 24-well plate format. The media used *ex vivo* is complete MEGM (Lonza). Continuous inhibitor treatment lasted between days 1-14. For late addition assays, assays with viable growth at day 6 or 8 (as observed using a light microscope) were divided to equal size groups (> 4 assays per treatment). Treatment with inhibitor or appropriate vehicle control lasted for subsequent 12 days. Inhibitors were added at concentrations as above every 3 days. In experiments represented in Figure 4, tumours (*n*=4) were allowed to grown for 7-14 d prior to inhibitor treatment lasting 3 additional days. Quantification of positive nuclei in invading cells was performed on entire sections obtained from the same tumour (>90 cells/preparation).

To terminate all experiments, cultures were fixed in 10% phosphate buffered formalin and wax embedded.

### Staining protocol of 3D *ex vivo* cultures for optical projection tomography

Large-scale specimen staining for optical projection tomography has followed a previously published protocol [4]. Briefly, fixed and permebialised preparations were incubated with primary antibody to identify the epithelial content using rabbit pan-cytokeratin (Cell Signalling*)*, and subsequently with a fluorescein-conjugated secondary antibody (Invitrogen). The preparation was mounted in 1% low melting agarose and dehydrated in 100% Methanol for 24 h. The specimen was cleared in BABB solution (1:2 Benzyl alcohol, Benzyl benzoate) for 24 h and scanned using the Optical Projection Tomograph (Bioptonics 3001M). Both target and auto-fluorescence were captured using a GFP+ filter and GFP1 filter respectively.

### Immunohistochemistry

Antigen retrieval for all staining was performed using sodium citrate buffer (18 μM Citric Acid, 82 μM sodium citrate, pH 6.0). Standard immunohistochemistry protocol was performed using the REAL EnVision mouse or rabbit kit (Dako), according to manufacturer's instructions. Primary antibodies used were: β-catenin, BD #610153, Mouse, 1:500; CD44, AbD serotec #MCA2504, Mouse, 1:10,000; Cleaved caspase-3, Cell Signaling #9661, 1:400; Cyclin D1, Dako #M3635, Rabbit 1:100 E-cadherin (intracellular), BD #610181, Mouse, 1:1500; G3BP2, Sigma #HPA018304, Rabbit, 1:5000; Ki67, Dako #M7240, Mouse, 1:400; phosphorylated STAT3 (Ser727), Eurogentec, #65367, Rabbit, 1:200-1:600; Rac1, GeneTex, #GTX100761, Rabbit, 1:100-1:200.

### Categorisation of effects on explant outgrowths *ex vivo*

After 14 d of treatment with either Rac inhibitior (EHT 1864) or STAT3 inhibitor (Stattic), all preparations were inspected using live microscopy. Cell invasion from the original tumour explant into the surrounding collagen is typically observed after 6-8 d from start of culture [5]. At each experimental time point, media was removed and cellular outgrowths ware determined by light microscopy (x10 magnification). The viability of outgrowths was confirmed subsequently using H&E sections from the fixed preparations.

## Supplementary Figures and Tables




