# Supplementary material for: Targeting of Rac GTPases blocks the spread of intact human breast cancer
Source: Oncotarget. 2012 Jun 9;3(6):608–19. doi: 10.18632/oncotarget.520 (PMC3442288; doi:10.18632/oncotarget.520)
Supplement: Supplementary file 1 [file oncotarget-03-608-s001.pdf]

Targeting of Rac GTPases blocks the spread of intact human breast cancer - Katz et al

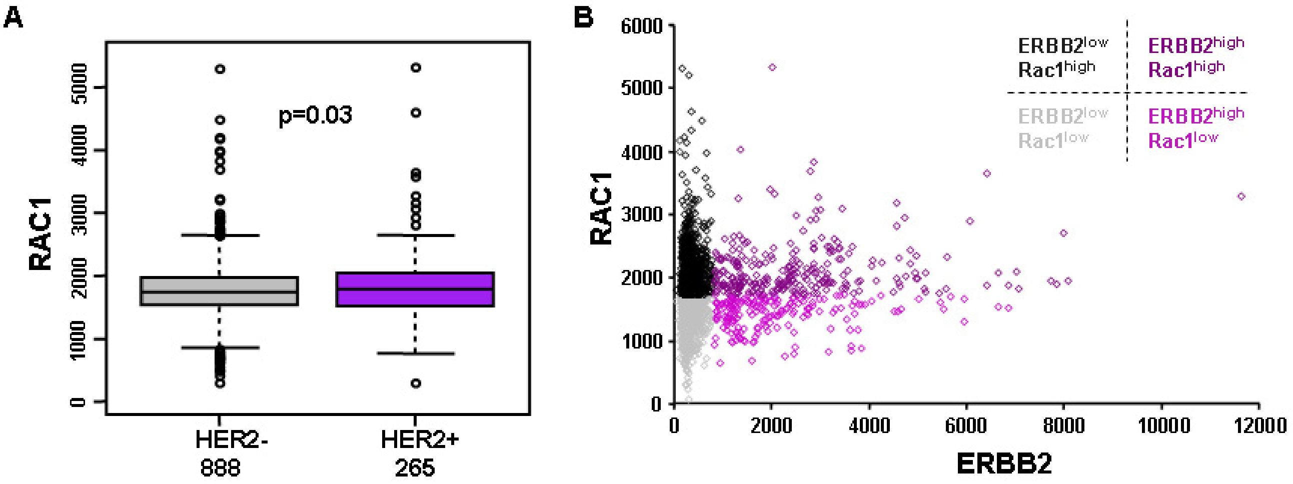

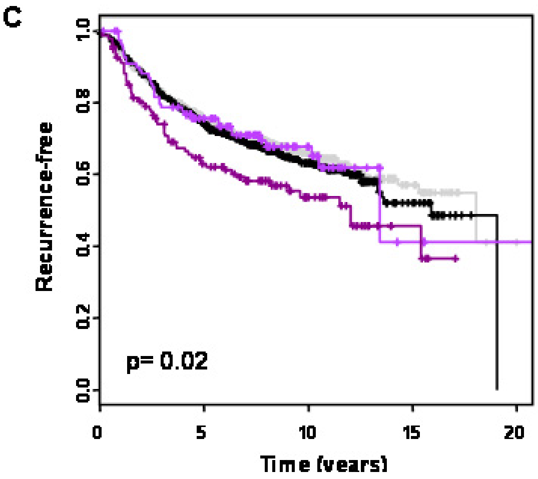

D

ERBB2^{low} RAC1^{low}	ERBB2^{low} RAC1^{high}	p=0.4
ERBB2^{low} RAC1^{low}	ERBB2^{high} RAC1^{low}	p=0.8
ERBB2^{low} RAC1^{high}	ERBB2^{high} RAC1^{low}	p=0.8
ERBB2^{low} RAC1^{low}	ERBB2^{high} RAC1^{high}	**p=0.003**
ERBB2^{low} RAC1^{high}	ERBB2^{high} RAC1^{high}	**p=0.006**
ERBB2^{high} RAC1^{low}	ERBB2^{high} RAC1^{high}	**p=0.04**

Figure S1: High RAC1 expression can sub-divide breast cancer patients with high HER2 gene expression into those with particularly poor prognosis. (A) *RAC1* gene expression levels are slightly elevated in HER2+ breast cancers in comparison to HER2- tumours ($p = 0.03$). HER2 status was determined by immunohistochemistry. *RAC1* expression levels were divided to low and high at the median. (B-D) Further analysis utilised *ERBB2* gene expression levels as surrogate measurement for *ERBB2* status: (B) Ranges of *RAC1* expression in *ERBB2*^{low} and *ERBB2*^{high} tumour groups were similar. Clinical outcomes of patients: (C) Kaplan-Meier survival curves for patients depending on their *ERBB2* and *RAC1* expression levels (color coding as in B). The trend was statistically significant ($p = 0.02$). (D) Statistically significant differences between patient groups ($p < 0.05$) are highlighted in bold.

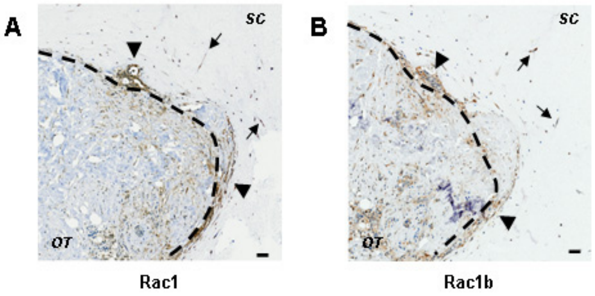

Figure S2: Rac1 isoforms are expressed in tumour outgrowths ex vivo. Immunohistochemistry showing that cells invading *ex vivo* express Rac1 protein (A) and its short isoform Rac1b (B). Rac1 and Rac1b are expressed in both tumour epithelial (*arrowheads*) and mesenchymal (*arrows*) invading cells. The dotted lines show border between original tumour explant (OT) and surrounding collagen (SC). Bars, 50 µm

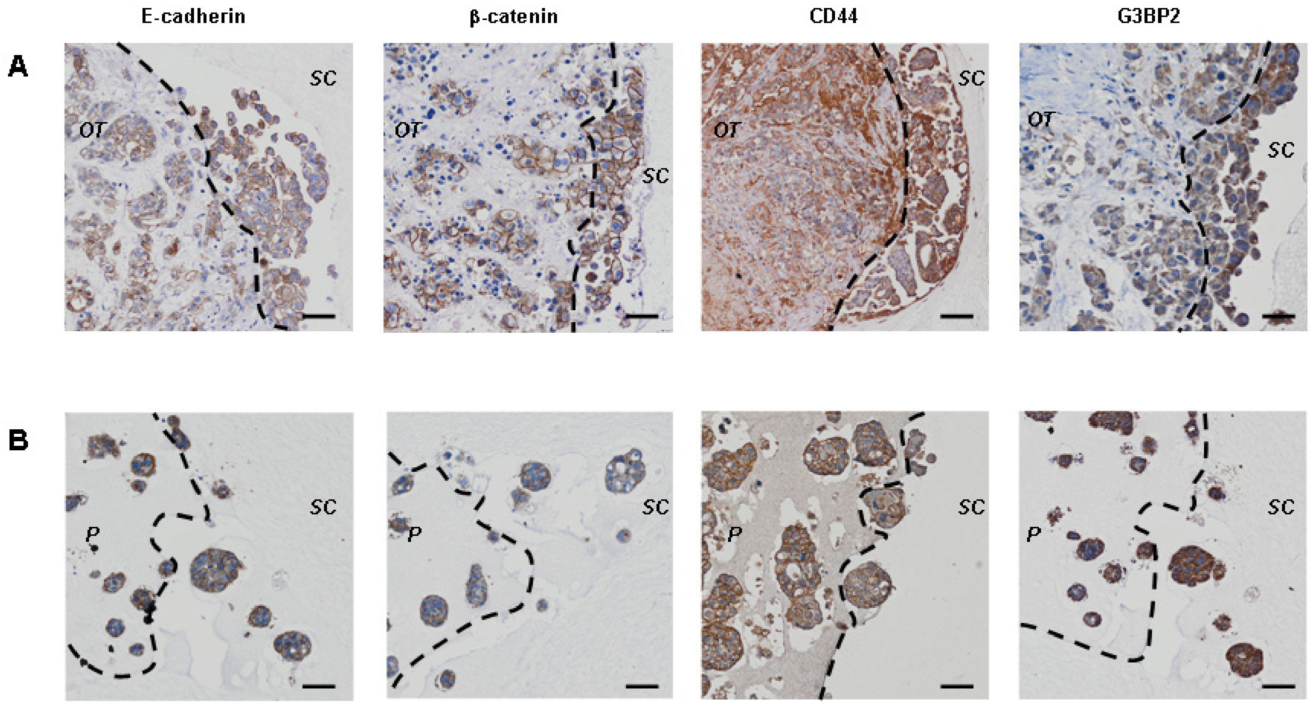

Figure S3: Similarities between ex vivo and cell line cultures. The collectively invading cells in tumour and cell line cultures display similar protein expression patterns. Representative images are shown for tumour (A) and cell line (B) invasion assays stained with antibodies for E-cadherin, beta-catenin, CD44 and G3BP2. Images are from a representative ER- HER2- tumour. The dotted lines show border between original tumour explant (OT) or (P) (A and B respectively) and surrounding collagen (SC). Bars, 50 µm.

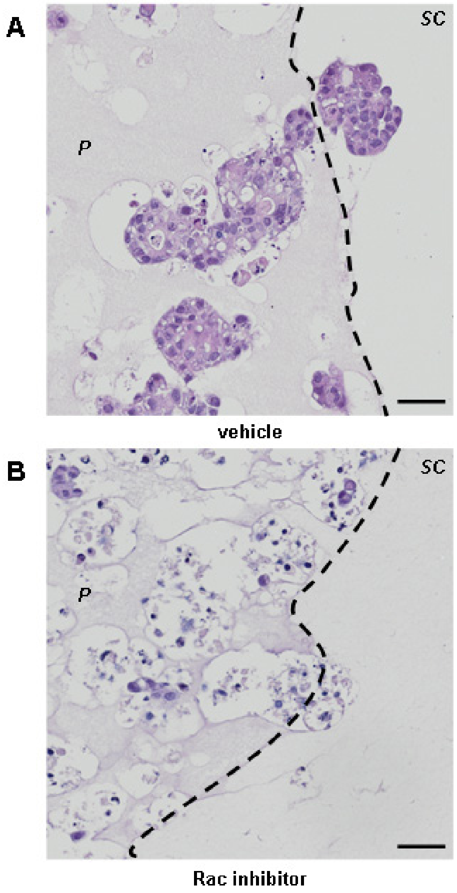

Figure S4: Cell line model of collective invasion. HCC1954 cell invasion into surrounding collagen after 10 d is shown using H&E staining (A). (B) Rac inhibition with EHT 1864 for 24 h results in blockage of invasion beyond the original plug and extensive cell death (see also Figure S6A). Shown here are representative images from a single experiment (*n*=6). The dotted lines show border between original cell line plug (P) and surrounding collagen (SC). Bars, top panels, 200 µm. bottom panels, 50 µm. Magnified areas (*bottom panels*) are outlined in black boxes (*top panels*).

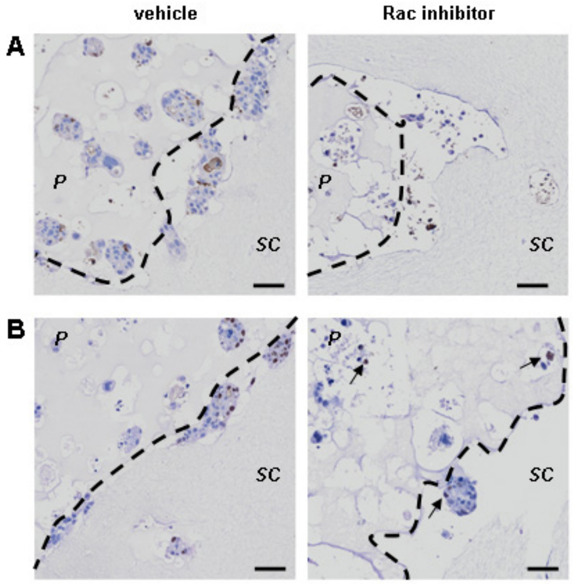

Figure S5: Rac inhibition blocks tumour cell line invasion and induces cell death. HCC1954 cell invasion into surrounding collagen after 10 d is shown using H&E staining (*left panels*), inhibited by EHT 1864 treatemnt in the last 24 h (*right panels*). Induction of cell death was detected by immunohistochemistry for cleaved caspase-3 (A). Proliferation, detected by Ki67, is still observed in the presence of EHT 1864 (B, arrows). Shown here representative images from a single experiment (*n*=6). The dotted lines show border between original cell line plug (P) and surrounding collagen (SC). Bars, 50 µm.

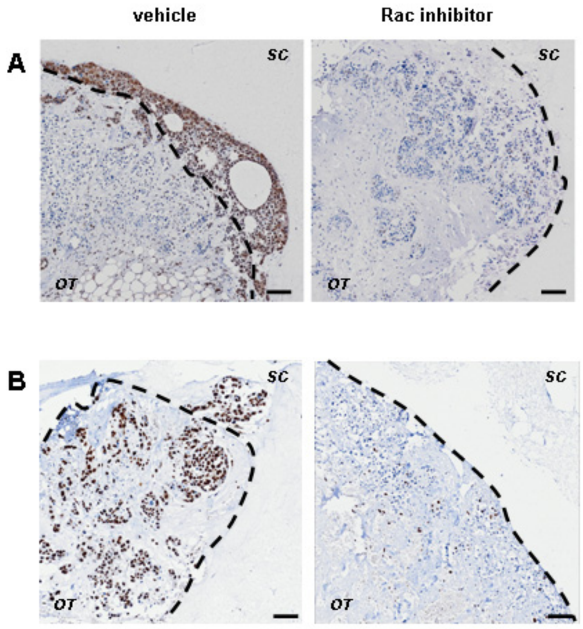

Figure S6: STAT3 activity is associated with invasion ex vivo and is down regulated by Rac inhibition. Rac inhibition with EHT 1864 (*right panels*; inhibition as in Figure 2) down-regulates *ex vivo* STAT3 Ser727 phosphorylation (A) and expression of STAT3 transcriptional target Survivin (B). The dotted lines show border between original tumour explant (OT) and surrounding collagen (SC). Bars, 50 µm.

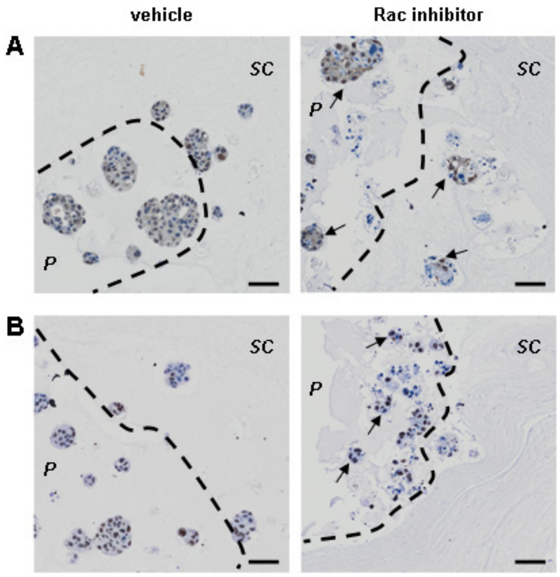

Figure S7: Rac inhibition blocks STAT3 activity. HCC1954 cell invasion into surrounding collagen after 10 d is shown using H&E staining (*left panels*), inhibited by EHT 1864 treatemnt in the last 24 h (*right panels*). Viable HCC1954 cells after Rac inhibition retain STAT3 phosphorylation (A, arrows) and Survivin expression (B, arrows).Shown here representative images from a single experiment (*n*=6). The dotted lines show border between original cell line plug (P) and surrounding collagen (SC). Bars, 50 µm.
